# Effect of chronic exogenous oxytocin administration on exercise performance and cardiovagal control in hypobaric hypoxia in rats

**DOI:** 10.1186/s40659-024-00573-3

**Published:** 2024-11-23

**Authors:** Camila Salazar-Ardiles, Carlos Cornejo, Cristobal Paz, Manuel Vasquez-Muñoz, Alexis Arce-Alvarez, Maria Rodriguez-Fernandez, Gregoire P. Millet, Mikel Izquierdo, David C. Andrade

**Affiliations:** 1 Exercise Applied Physiology Laboratory, Centro de Investigación en Fisiología y Medicina de Altura (FIMEDALT), Departamento Biomedico, Facultad de Ciencias de la Salud, Universidad de Antofagasta, Antofagasta, Chile; 2 Navarrabiomed, Hospital Universitario de Navarra (UHN), Universidad Pública de Navarra (UPNA), IdiSNA, Pamplona, Navarra, Spain; 3 Dirección de Docencia de Especialidades Médicas, Dirección de Postgrado, Facultad de Medicina y Ciencias de la Salud, Universidad Mayor, Santiago, Chile; 4 Institute for Biological and Medical Engineering, Schools of Engineering, Medicine, and Biological Sciences, Pontificia Universidad Católica de Chile, Santiago, Chile; 5 Institute of Sport Sciences, University of Lausanne, Lausanne, CH-1015 Switzerland; 6 Escuela de Kinesiología, Facultad de Odontología y Ciencias de la Rehabilitación, Universidad San Sebastian, Santiago, Chile

**Keywords:** Parasympathetic, Autonomic control, High-altitude, Baroreflex

## Abstract

**Background:**

Outstanding exercise performance has been associated with an exacerbated vagal outflow. Nevertheless, during high-altitude hypobaric-hypoxia (HH), there is a baroreflex-dependent parasympathetic withdrawal and exercise performance deterioration. Notably, vagal control is pivotal in exercise performance, and exogenous oxytocin (OXY) administration has been shown to enhance parasympathetic drive; however, no evidence shows their role in exercise performance during HH. Then, this study aimed to examine the effect of prolonged exogenous oxytocin (OXY) administration on exercise performance during hypobaric hypoxia (HH) in rats.

**Results:**

A vehicle group (*n* = 6) and an OXY group (*n* = 6) performed incremental exercise and baroreflex tests during both normobaric normoxia (NN) and HH (PO_2_: 100 mmHg, simulated 3,500 m) prior (pre-) and after (post-) 14 days of administration. The results showed that at pre-, there were no significant differences in exercise performance between the two groups, while at post-, the OXY group exhibited similar performance between NN and HH, while the Vehicle group maintained a significant decline in performance at HH compared to NN. At post-, the Vehicle group also demonstrated a reset in the baroreflex and a worse bradycardic response in HH, which was reversed in the OXY group, while the hypoxic ventilatory response was similar in both groups.

**Conclusion:**

The findings suggest prolonged OXY administration prevents impaired exercise performance and vagal control during short-term HH.

## Background

Approximately 81.6 million individuals reside at elevations over 2,500 m, and 14.4 million reside at 3,500 m or higher [1]. In response to reduced O_2_ availability, hyperventilation and autonomic imbalance have been observed, primarily associated with elevated metabolic demand [2, 3]. Indeed, exposure to high-altitude hypobaric hypoxia (HH) promotes impairment of baroreflex (BR) control, characterized by severe BR-dependent parasympathetic withdrawal after 24 h of HH exposure [4]. Nevertheless, one of the most evident effects of HH is its impact on human physical performance. Furthermore, it has been well documented that acute exposure (less than 24 h) to HH decreases VO_2_max [5, 6] and endurance capability [7–9].

The autonomic nervous system is divided into sympathetic and parasympathetic arms [10]. Notably, outstanding exercise performance has been associated with an exacerbated vagal outflow [11, 12] through increased heart rate reserve [13]. However, counterintuitively, it has been believed that parasympathetic drive progressively decreases concomitant with a sympathoexcitation during exercise [14]. Contrary to this belief, studies in rats have shown that activation of parasympathetic neurons through optogenetic and chemogenetic approaches can improve exercise performance, mimicking the effects of a training regimen, while silencing cardiovagal neurons can impair exercise capacity [15]. Further, it has been shown that parasympathetic drive is crucial to exercise-mediated increases in coronary artery blood flow through vasoactive intestinal peptide (vasodilator) in sheep [16]. Interestingly, severe deterioration of parasympathetic drive is associated with a parallel decline in exercise performance during hypoxia in both humans and rodents [4, 8, 17]. While it is well established that exercise deterioration is closely linked to parasympathetic withdrawal [15, 16] and that this is a crucial factor in physical performance [11, 12], it is possible that parasympathetic modulation could also influence exercise performance in HH.

Oxytocin (OXY) is thought to enhance parasympathetic outflow through cardio-vagal oxytocinergic neurons [18]. These neurons reside in the paraventricular nucleus (PVN) of the hypothalamus [19] and project to the nucleus of the solitary tract (NTS)/dorsal motor nucleus of the vagus (DMNV) area [20–22]. The projections of these neurons facilitate vagal outflow to the heart during pressure challenges, thereby maintaining the magnitude and gain of BR-mediated bradycardia [23]. Oxytocin has been suggested to be a sports hormone [24]; however, there is no evidence of whether it is possible to improve/maintain exercise performance in HH, considering that there is a parasympathetic modulation and performance impairment in this environment. Therefore, we investigated the impact of prolonged exogenous OXY administration on physical performance in HH. We tested the hypothesis that OXY administration mitigates the performance impairment in HH and promotes increased parasympathetic cardiovagal control.

## Materials and methods

### Animals

Adult male Wistar Kyoto rats (*n* = 12, 276±5 g) were randomly divided into two groups: the Vehicle group (Veh, *n* = 6) and the OXY group (*n* = 6). The Veh group received NaCl 0.9% solution (200 µL, administered intraperitoneally). In contrast, the OXY group received OXY (1 mL, 5 U.I. in NaCl 0.9%, Laboratorio Sanderson, Ñuñoa, Chile) (0.433 µg/kg, 200 µL, administered intraperitoneally) once daily, for 14 days. Both animal groups increased their body weight after 14 days, which was not associated with OXY administration (Veh: 281 ± 8 vs. 298 ± 8 g, pre vs. post, *p* = 0.003) (OXY: 264 ± 8 vs. 278 ± 12 g, pre vs. post, *p* = 0.006). The animals were sourced from the animal facility at the Universidad de Antofagasta and were housed under controlled temperature and humidity conditions with a standard 12-hour light/dark cycle. The rats had unrestricted access to water and a standard diet (Prolab RMH3000; LabDiet, USA).

Animal welfare guidelines for this study were established by the American Physiological Society. The protocols were approved by the Ethics Committee on Scientific Research at the Universidad de Antofagasta (CEIC-UA 438/2022). At the end of the experiments, all animals were humanely euthanized with an overdose of sodium pentobarbital (100 mg/kg, i.p.).

### Surgical and experimental procedures

**Fig. 1:**
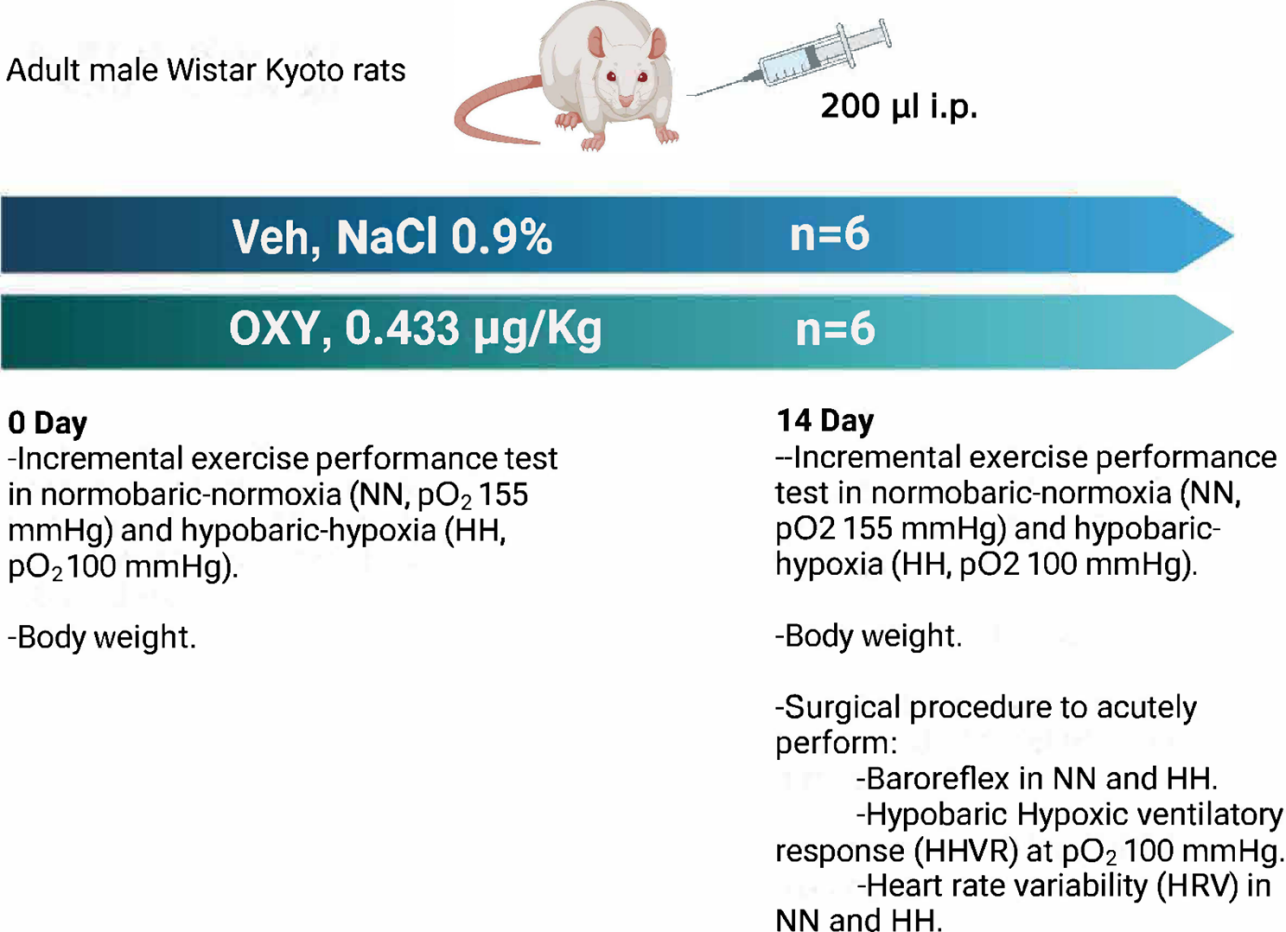
Experimental design

The experimental design is illustrated in Fig. 1. Before (pre-, 2 days prior to beginning treatments) and after (post-, at days 13 and 14) the Veh and OXY administration period, the exercise performance was assessed using an incremental exercise test under both NN and HH conditions (Fig. 5A). Afterward, the animals were subjected to physiological monitoring (heart rate variability (HRV), baroreflex, and hypobaric hypoxic ventilatory response (HHVR)) at the end of the experiment, similar to those previously described [4, 25]. At the endpoint (14 days after Veh or OXY administration), the rats were anesthetized using 40 mg/kg and 1 g/kg α-chloralose and urethane (Sigma-Aldrich), respectively. Once deeply anesthetized, they were positioned supine, maintaining their body temperature at 38.0 ± 0.5 °C through a controlled warming mat (Kent Scientific model RT-0515). A flexible tube was inserted into the trachea to monitor airflow and was connected to a pneumotachograph to study the HHVR. Subsequently, a catheter was placed in the jugular vein (PE50 polyethylene tubing containing a saline solution) for drug administration, and another catheter (PE-10 connected to PE-50 tubing filled with a heparin saline solution, concentration: 5 I.U.) was placed in the left femoral artery to measure blood pressure (BP) (PowerLab/4SP, ADInstruments, Castle Hill, NSW, Australia).

### Heart rate variability (HRV)

Heart rate variability (HRV) indirectly indicates the autonomic balance in the heart [10, 25, 26]. The R-R time series was calculated from dP/dt from a 10-minute BP recording. By applying an autoregressive algorithm following Hann windowing with a 50% overlap, the power spectral density of the HRV was derived. Low-frequency HRV (LF_HRV_), range: 0.04–0.6 Hz; and high-frequency HRV (HF_HRV_), range: 0.6–2.4 Hz [25]. In addition, the LF_HRV_-to-HF_HRV_ ratio (LF/HF_HRV_) was employed as a global autonomic index. LF_HRV_ and HF_HRV_ are reported in normalized units (n.u.). We used spectral non-stationary analysis with a 2-second resolution to evaluate the short-term variability. This analysis was performed with Kubios HRV Premium Software V 3.1 (Kubios, Finland).

### Baroreflex function

The BR sensitivity was assessed using serial doses (50 µL) of sodium nitroprusside (SNP, 0.4, 0.8, 1.6, 3.2, 6.4, 12.8, and 25.6 µg/kg; Sigma-Aldrich, United States) and phenylephrine (PHE, 0.2, 0.4, 0.8, 1.6, 3.2, 6.4, and 12.8 µg/kg; Sigma-Aldrich, United States). These drugs were administered to facilitate a decrease (SNP) or increase (PHE) in BP, with subsequent HR changes. SNP and PHE injections were administered in a dose-dependent manner, from lowest to highest. The cardiac BR function was analyzed using logistic regression across the entire pressure range [4, 27–29]. The data were adjusted to the equation: FC = A / [1 + exp (B (SBP-C))] + D, where A is the range of HR, B is the slope coefficient, C is the pressure at the midpoint of the range (BP_50_), and D is the minimum HR. The maximum slope (maximum gain) was determined by the first derivative of the baroreflex curve and calculated using the equation gain = A1 × A2 × [1/4], where A1 is the range and A2 is the average slope. Further HR at the midpoint of the range (HR_50_) was calculated. All analyses were performed using the GraphPad Prism software (version 10.0; La Jolla, CA, USA) and Excel.

### Blood pressure

The BP assessment was performed in the left femoral artery. The BP signal was recorded using an analog-digital system (PowerLab/4SP, AD Instruments, Castle Hill, NSW, Australia) and analyzed using LabChart 7.0 software (AD Instruments, Castle Hill, NSW, Australia). From the systolic blood pressure (SBP) and diastolic BP (DBP) raw signals, the pulse pressure (PP = SAP-DAP) and mean arterial blood pressure (MABP = 1/3 of SAP + 2/3 of DAP) were calculated. Heart rate (HR) was calculated from the first derivative of BP (dP/dt) [25, 26].

### Hypobaric-hypoxic ventilatory response (HHVR)

Initially, the animals were maintained under normobaric normoxia (NN: O_2_ partial pressure [PO_2_], 155 mmHg), and the baseline breathing was assessed during 10 min recording. Subsequently, the rats were subjected to HH (PO_2_: 100 mmHg; speed decay: 1.57 mmHg/min) environmental conditions in a hypobaric hypoxic chamber (Genetic Core, Peru) simulating 3,500 m (speed ascend: 100 m/min). The breathing at HH was assessed during a 10-minute recording. To determine HHVR, the animals were anesthetized inside the hypobaric hypoxic chamber. After deep anesthesia, a catheter was placed in the trachea and connected to a spirometer pod (PowerLab/4SP, ADInstruments, Castle Hill, NSW, Australia), which was previously calibrated. From ventilatory flow, the minute ventilation (tidal volume [V̇_T_] ⋅ respiratory frequency [R_f_] = V̇_E_) was calculated [30]. HHVR was expressed as a percentage difference between hypoxia and normoxia (% of normoxia). The breathing signal was analyzed using LabChart 7.0 software (AD Instruments, Castle Hill, NSW, Australia).

### Incremental exercise test

Before and after Veh or OXY administration, a progressive exercise test was used to assess the exercise capacity of all animals, as previously described [31]. Before the exercise test, the animals were familiarized with the treadmill (LE 8700 TS model, Panlab Harvard Apparatus, Spain) for 3 min, and after that, started to run at 16 cm/s without inclination. This acclimatization period was for one week. After the familiarization period, the rats began running at 41.7 cm/s with a 2º incline; the treadmill speed was increased by 5 cm/s every minute with a constant inclination until exhaustion. A mild electrical grid (0.5 mA) was used to motivate exercise at the back of the treadmill. The test concluded when the rats displayed signs of exhaustion, defined as remaining on the electrical grid for 10 s without being able to continue or keep up with the treadmill’s pace (Fig. 5). The work done until exhaustion was expressed in Joules (J).

### Statistical analysis

Data are expressed as mean ± standard error of the mean (S.E.M.) in the results section, and median ± min-max in the figures, except for exercise performance, which is shown as mean ± standard deviation (S.D.). The normality of the data was assessed using the Shapiro-Wilk test, and Levene’s test determined the homoscedasticity of the variance. Differences between groups were evaluated using repeated measures ANOVA (2 × 2) followed by Holm-Sidak posthoc. The cardiorespiratory responses to HH were analyzed with a non-parametric Mann-Whitney test. In addition, the statistical power (1-β) (SP) was calculated for every significant comparison (G*Power, Germany). Differences were considered statistically significant at *P* < 0.05. All analyses were performed using the GraphPad Prism software (version 10.0, La Jolla, CA, USA).

## Results

### Effects of chronic administration of OXY on heart rate variability in NN and HH

**Fig. 2:**
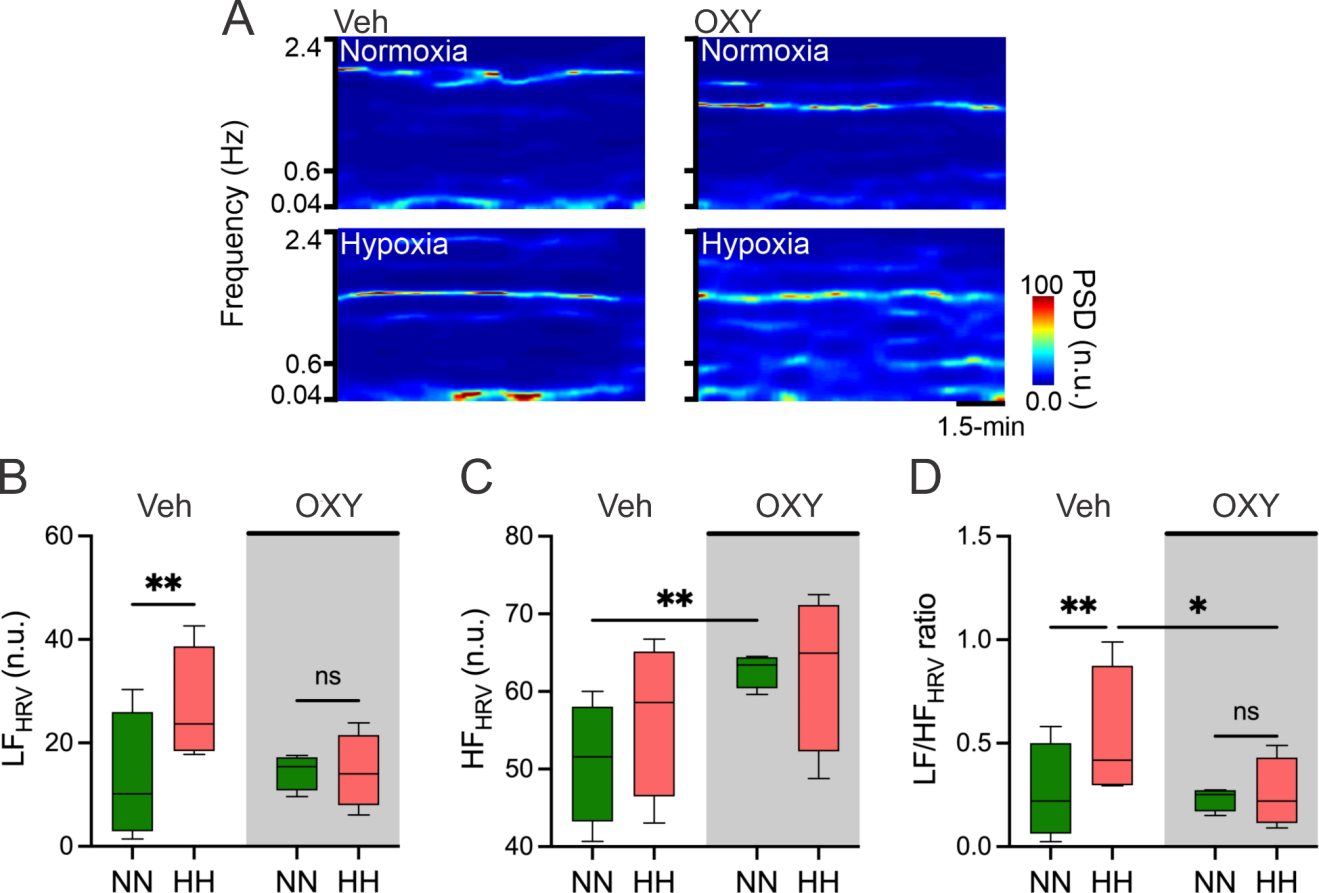
Effects of OXY administration on rats’ heart rate variability (HRV) during normobaric-normoxia (NN) and hypobaric-hypoxia (HH). (**A**) Representative power spectral spectrum density during NN and HH after 14 days of Veh and OXY administration. (**B**) Low-frequency HRV (LF_HRV_) component. (**C**) High-frequency HRV (HF_HRV_) component. (**D**) LF/HF_HRV_ ratio. *N* = 6 for each group (repeated measures ANOVA (2 × 2) followed by Holm-Sidak posthoc. *, *p* < 0.05; **, *p* < 0.01)

The Veh group exhibited a significant increment of the LF_HRV_ (26.9 ± 5.6 vs. 13.0 ± 6.2 n.u., HH vs. NN, SP: 0.39) (Fig. 2A and B) and LF/HF_HRV_ ratio (0.53 ± 0.2 vs. 0.26 ± 0.1 n.u., HH vs. NN, SP: 0.15) (Fig. 2A and D) during HH compared to NN. The OXY administration prevents the increment of the LF_HRV_ (14.5 ± 3.6 vs. 14.4 ± 1.7 n.u., HH vs. NN) (Fig. 2A and B) and LF/HF_HRV_ ratio (0.25 ± 0.1 vs. 0.23 ± 0.1 n.u., HH vs. NN) (Fig. 2A and D) during HH compared to NN. In addition, the LF/HF_HRV_ ratio was significantly reduced after OXY administration compared to Veh animals during HH (0.23 ± 0.1 vs. 0.53 ± 0.2 n.u., Veh vs. OXY, SP: 0.17) (Fig. 2D). Further, OXY significantly increased the HF_HRV_ during NN compared to the Veh group (62.7 ± 1.1 vs. 50.9 ± 3.9 n.u., OXY vs. Veh, SP: 0.55) (Fig. 2C).

The Veh group showed no significant differences in SBP, DBP, MABP, and HR during NN compared to HH; however, OXY administration elicited a significant decrease in SBP (*p* = 0.026; SP: 0.31) and PP (*p* = 0.037; SP: 0.10) during HH compared to NN (Table 1). Moreover, we analyzed the Δ effects of HH stimulus between groups. We found that OXY significantly decreased ΔSBP (*p* = 0.008; SP: 0.34), ΔPP (*p* = 0.026; SP: 0.44), and ΔHR (*p* = 0.041; SP: 0.25) compared to the Veh group (Table 1).

**Table 1:** Cardiorespiratory variables during NN and HH in Veh and OXY animals

	Veh (*n* = 6)		OXY (*n* = 6)		ΔHH test (HH–NN)	
	NN	HH	NN	HH	Veh (*n* = 6)	OXY (*n* = 6)
SBP (mmHg)	74.5 ± 10.8	79.5 ± 8.8	85.8 ± 6.5	68.7 ± 5.7*	4.9 ± 3.1	-17.1 ± 9.9^+^
DBP (mmHg)	42.3 ± 3.6	44.6 ± 3.7	62.2 ± 11.4	50.0 ± 9.6	2.2 ± 4.4	-12.5 ± 10.9
MABP (mmHg)	52.9 ± 5.5	56.1 ± 3.5	70.0 ± 9.5	55.9 ± 8.2	3.1 ± 3.9	-14.0 ± 10.5
PP (mmHg)	32.2 ± 8.6	34.9 ± 10.1	23.6 ± 6.6	18.7 ± 4.5*	2.7 ± 1.6	-4.6 ± 2.6^+^
HR (beats/min)	246.6 ± 18.0	269.2 ± 22.1	297.4 ± 14.4	226.1 ± 25.6	2.5 ± 29.2	-71.4 ± 28.3^+^
V̇_T_ (mL)	0.37 ± 0.08	0.48 ± 0.01	0.44 ± 0.13	0.55 ± 0.15*	0.11 ± 0.12	0.10 ± 0.03
R_f_ (breaths/min)	82.7 ± 6.7	94.9 ± 7.3*	87.7 ± 15.8	99.5 ± 16.1*	12.1 ± 0.7	11.7 ± 0.2
V̇_E_ (mL/min)	30.2 ± 6.8	45.1 ± 11.4*	33.0 ± 7.5	46.3 ± 9.5*	15.0 ± 2.6	13.5 ± 8.3

Data are shown as mean ± standard error of the mean (SEM). NN: normobaric normoxia (PO_2_: 155 mmHg); HH: hypobaric hypoxia (PO_2_: 100 mmHg); Veh: vehicle group; OXY: oxytocin group; SBP: systolic blood pressure; DBP: diastolic blood pressure; MABP: mean arterial blood pressure; PP: pulse pressure; HR: heart rate; V̇_T_: tidal volume; R_f_: respiratory frequency; and V̇_E_: minute ventilation. ΔHH test (HH–NN): the difference between HH and HH. The data was analyzed using repeated measures ANOVA (2 × 2) followed by Holm-Sidak posthoc. The pairs comparison (ΔHH test) was analyzed using the Mann-Whitney test. *, *p* < 0.05, HH vs. NN; ^+^, *p* < 0.05, OXY vs. Veh

### Effect of OXY administration on BR control in rats during NN and HH

**Fig. 3:**
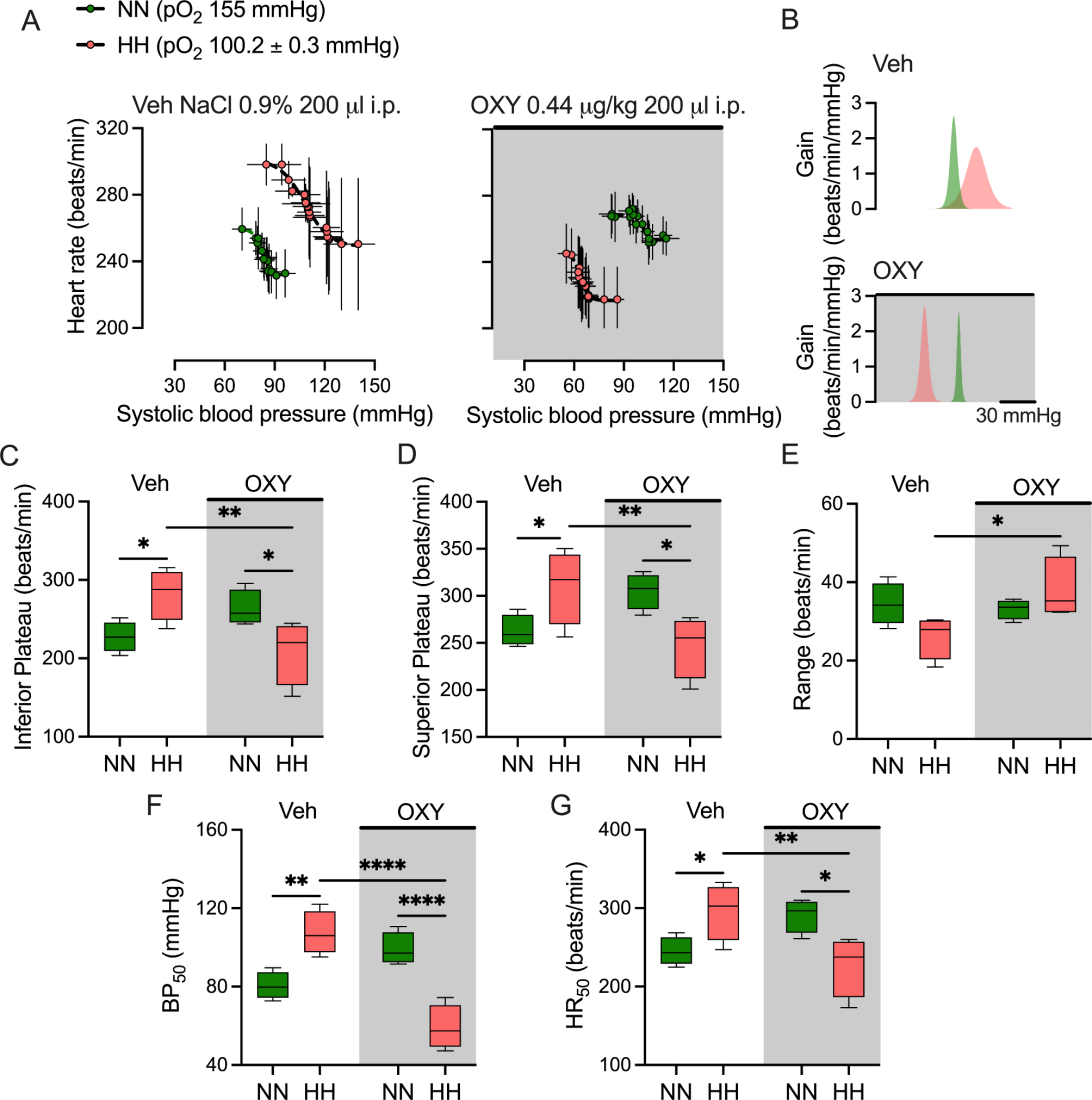
Effects of OXY administration on rats’ baroreflex control during normobaric-normoxia (NN) and hypobaric-hypoxia (HH). (**A**) Baroreflex curve and (**B**) peak gain during NN and HH after 14 days of Veh and OXY administration. (**C**) Inferior plateaus. (**D**) Superior plateaus. (**E**) Heart rate range (superior plateau - inferior plateau). (**F**) Blood pressure 50 (BP_50_). (**G**) Heart rate 50 (HR_50_). *N* = 6 for each group (repeated measures ANOVA (2 × 2) followed by Holm-Sidak posthoc. *, *p* < 0.05; **, *p* < 0.01; ***, *p* < 0.001; ****, *p* < 0.0001)

After 14 days of OXY administration, baroreflex was assessed during NN and HH (Fig. 3). The BR was markedly reset in HH compared to NN in the Veh group but not in the OXY group (Fig. 3A), without differences in the peak gain of the baroreflex function (Fig. 3B). Indeed, the inferior (282.3 ± 16.3 vs. 227.4 ± 9.8 beats/min, *p* < 0.05, SP: 0.46, HH vs. NN, Fig. 3C) and superior plateaus (310.3 ± 19.8 vs. 262.5 ± 8.4 beats/min, *p* < 0.05, SP: 0.35, HH vs. NN, Fig. 3D) were increased parallel to the enhancement of BP_50_ (107.3 ± 5.6 vs. 80.5 ± 3.5 mmHg, *p* < 0.01, SP: 0.81, HH vs. NN, Fig. 3F) and HR_50_ (296.3 ± 18.0 vs. 244.9 ± 9.0 beats/min, *p* < 0.05, SP: 0.47, HH vs. NN, Fig. 3G) in HH compared to NN in the Veh group. Conversely, OXY rats display contrary effects, significantly decreasing inferior (209.1 ± 20.5 vs. 263.7 ± 11.4 beats/min, *p* < 0.05, SP: 0.41, HH vs. NN, Fig. 3C) and superior plateaus (247.1 ± 16.6 vs. 305.2 ± 9.6 beats/min, *p* < 0.05, SP: 0.50, HH vs. NN, Fig. 3D), BP_50_ (59.1 ± 5.7 vs. 99.1 ± 4.2 mmHg, *p* < 0.0001, SP: 0.97, HH vs. NN, Fig. 3F), and HR_50_ (227.1 ± 19.3 vs. 291.0 ± 10.7 beats/min, *p* < 0.05, SP: 0.54, HH vs. NN, Fig. 3G) in HH compared to NN. Moreover, the OXY administration substantially reduced inferior (209.1 ± 20.5 vs. 282.3 ± 16.3 beats/min, *p* < 0.01, SP: 0.62, OXY vs. Veh, Fig. 3C) and superior plateaus (247.1 ± 16.6 vs. 310.3 ± 19.8 beats/min, *p* < 0.01, SP: 0.39, OXY vs. Veh, Fig. 3D), BP_50_ (59.1 ± 5.7 vs. 107.3 ± 5.6 mmHg, *p* < 0.0001, SP: 0.98, OXY vs. Veh, Fig. 3F), HR_50_ (227.1 ± 19.3 vs. 296.3 ± 18.0 beats/min, *p* < 0.01, SP: 0.48, OXY vs. Veh, Fig. 3G). Moreover. the baroreflex range in HH was larger (37.9 ± 3.9 vs. 26.2 ± 2.7 beats/min, *p* < 0.05, SP: 0.41, Fig. 3E) in the OXY than in the Veh group.

### Effects of OXY on HHVR and exercise performance

**Fig. 4:**
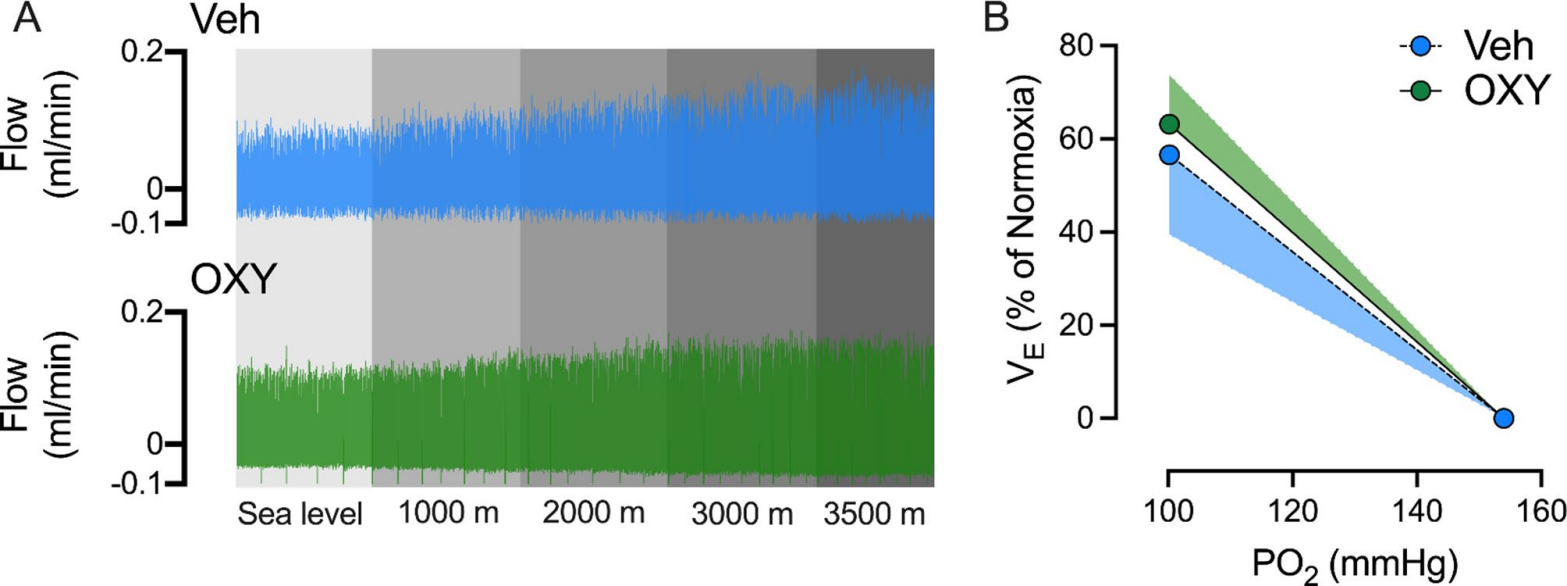
Hypobaric-hypoxic ventilatory response (HHVR) (**A**) Representative traces of ventilatory flow from one Veh rat and one OXY rat (left panel) during normobaric normoxia (NN) (sea level, pO2 155 mmHg) and hypobaric hypoxia (HH). (**B**) Both animal groups display similar HHVR without significant differences (right panel) (*n* = 6 for each group)

The OXY administration had no significant difference in HHVR since there was no difference between the OXY and the Veh groups (63.1 ± 10.8 vs. 56.6 ± 17.2% of NN, *p* > 0.05, Fig. 4A and B). However, in both groups, HH elicited a significant increment of R_f_ (Veh, SP: 0.14) (OXY, SP: 0.10) and V̇_E_ (Veh, SP: 0.15) (OXY, SP: 0.13), compared to NN. The V̇_T_ was only increased in the OXY group (SP: 0.10) (Table 1). Further, we analyzed the Δ effects of HH stimulus between groups. We do not find significant differences between groups (Table 1).

**Fig. 5:**
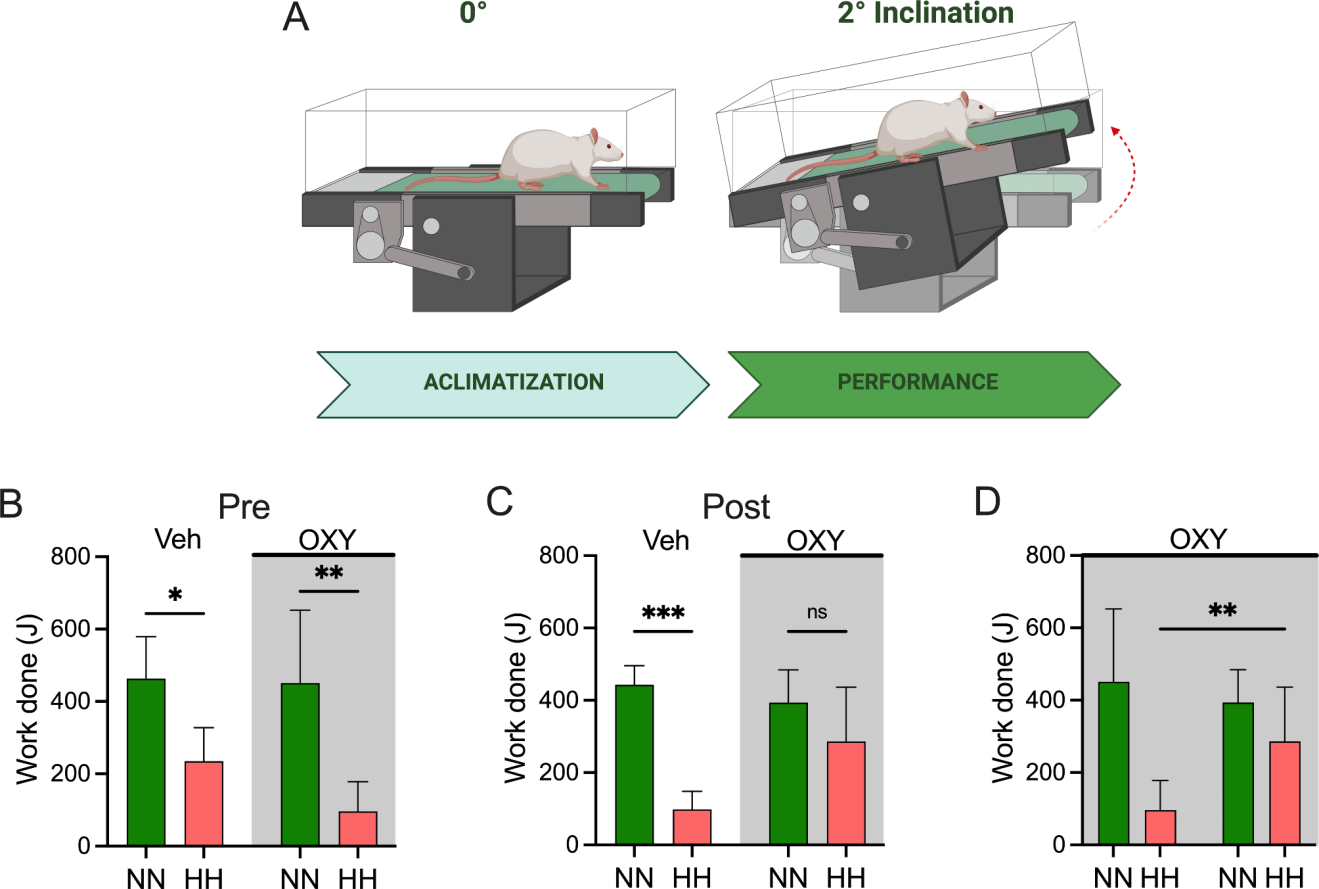
Effects of OXY administration on exercise performance during normobaric-normoxia (NN) and hypobaric-hypoxia (HH) in rats. (**A**) A representative cartoon depicts the exercise training test protocol. (**B**) Before OXY administration, the animal showed similar exercise deterioration during HH compared to the NN. (**C**) Notably, 14 days of OXY administration promoted a non-impairment of exercise performance during HH, which was observed in the Veh group. (**D**) OXY achieved to improve the work done during HH (repeated measures ANOVA (2 × 2) followed by Holm-Sidak posthoc. *, *p* < 0.05; **, *p* < 0.01; ***, *p* < 0.001) (*n* = 6 for each group)

Exercise performance was assessed at pre- and post- in both NN and HH (Fig. 5A). Before Veh or OXY administration (Pre-), both groups showed similar work decline in HH compared to NN (Veh: 234.9 ± 92.7 vs. 463.2 ± 116.1 J, *p* = 0.028, SP: 0.74; OXY: 96.5 ± 81.6 vs. 451.2 ± 200.9 J, *p* = 0.002, SP: 0.84, Fig. 5B). Fourteen days of OXY prevented the exercise performance impairment in HH (286.3 ± 149.8 vs. 393.7 ± 90.7 J, *p* = 0.089), which was not observed with Veh administration (98.4 ± 50.3 vs. 442.9 ± 52.6 J, *p* = 0.001, SP: 0.64) (Fig. 5C). Furthermore, in the OXY group, the work done was significantly improved from pre- to post- in HH (96.5 ± 81.6 vs. 286.3 ± 149.8 J, *p* = 0.004, SP: 0.37) (Fig. 5D).

## Discussion

The present study aimed to investigate the effects of prolonged exogenous OXY administration on physical performance during acute exposure to HH. The study’s principal findings are that (i) OXY was able to prevent the HH-dependent deterioration of work done; (ii) OXY administration prevented autonomic control impairment (parasympathetic withdrawal) during HH; ii) HH-dependent BR resetting was reversed after OXY administration without significant effects on HHVR. These findings confirm that OXY administration prevented both HH-dependent exercise performance impairment and autonomic control impairment elicited by a hypoxic environment.

### Role of OXY in exercise capacity during HH

It has been well documented that VO_2_max, exercise performance, and functional capacity are severely compromised in humans [3, 5, 7, 32–34] and rats [35, 36] during exposure to HH, which has been determined through different exercise tests between humans and animals, which could be a limitation in transferring the knowledge from preclinical models to a human setting. Indeed, it has been estimated that VO_2_max decreases by 6.3% per 1,000 m increasing altitude [6]. The decrease in exercise performance is mainly due to the hypoxic environment [37]; however, it has also been shown that activation or silencing of cardiovagal neurons can either improve or deteriorate exercise performance, respectively [15]. Therefore, it is possible to speculate that parasympathetic withdrawal precedes exercise performance deterioration in HH. Our data indicate that prior to OXY administration, exercise capacity was altered in HH in both groups; however, this was prevented (or at least blunted) after 14 days of exogenous OXY administration. This could be through increased heart rate reserve [13] as well as exercise-mediated increases in coronary artery blood flow through the release of vasoactive intestinal peptides in the heart [16]. Nevertheless, to our knowledge, this is the first time that OXY administration was shown to mitigate hypoxia-induced exercise performance impairment. This may be due at least partly to improved parasympathetic control, as OXY mediates their activation. However, our results fail to determine this relationship because our data do not necessarily show a cause-and-effect relationship between autonomic functions and exercise performance during HH. Nevertheless, these findings open the door for further investigation into the potential role of OXY as a doping agent, which may be of interest to the World Anti-doping Agency [38].

### Role of OXY in autonomic control and BR function in high-altitude HH

Exposure to high-altitude is commonly reported to induce autonomic control impairment and BR withdrawal [4, 39]. Although several conditions, including HH exposure modify cardiovagal BR [40–42], the underlying mechanisms remain poorly understood [4, 43]. Nevertheless, chronic intermittent hypoxia [26, 44–47], high altitude [4, 41, 42, 48], and suffocation [49], in most cases [50], promote autonomic control impairment and BR deterioration. Indeed, here we show that acute HH promotes autonomic imbalance, as assessed by HRV and BR resetting; however, exogenous OXY administration markedly reversed these effects. Mechanistically, OXY has been proposed to increase parasympathetic outflow through cardiovagal oxytocinergic neurons [18] allocated to the PVN of the hypothalamus [19]. These neurons facilitate the parasympathetic drive to the heart, preserving the magnitude and gain of baroreceptor-mediated bradycardia [23]. Intranasal OXY administration has improved autonomic control, determined through HRV, in obstructive sleep apnea patients [51]. Furthermore, OXY was not only able to improve autonomic control but also enhance breathing patterns and reduce apnea duration in these patients [51, 52]. Therefore, established evidence strongly shows that OXY could help improve and maintain autonomic control and BR function during acute exposure to HH. However, the present study failed to determine the precise mechanism associated with exogenous OXY administration. Further studies must focus on the cellular and molecular mechanisms related to increased parasympathetic drive from cardiovagal neurons in HH.

### Role of OXY in the hypobaric-hypoxic ventilatory response (HHVR)

One of the principal and most-known short-term responses to HH is an increase in ventilation facilitated by peripheral chemoreceptors. The principal peripheral chemoreceptors are the carotid body (CB), composed of glomus or type I cells and sustentacular cluster type II cells [53–55]. It has been proposed that chemoreceptors and baroreceptors share central autonomic nuclei [56], where chemoreceptors are hierarchically over baroreceptors in hypoxia [26, 57]. Accordingly, our data show that the increment in ventilation coincides with baroreflex resetting during exposure to HH. Indeed, we found that at a PO_2_ of 100 mmHg, minute ventilation increased concomitantly with an increment of the inferior and superior plateaus, BP_50,_ and HR_50_ of the BR function during HH in the control group. These data suggest that activation of peripheral chemoreceptors modifies BR function. Moreover, we observed that OXY administration reversed BR control without significant effects on HHVR. Overall, these results indicate that OXY did not modify HH-dependent CB activity. The BR-related effects could be associated with efferent activity and/or directly in the tissue.

### Possible adverse effects of OXY

OXY administration is not free of secondary effects. Indeed, it has been documented that OXY can propitiate postpartum bleeding problems [58], decrease BP [59], restrain food consumption [60], and, therefore, body weight alterations, as well as problems during pregnancy. Our data revealed that OXY animals increased BP during normoxia, which is not according to previous evidence and could be related to the doses used in the present study. Nevertheless, our data also revealed that the BP decreased during HH. In addition, although weight loss has been documented [60], we didn’t observe these events after 14 days of OXY administration. We observed an increment of body weight in both groups, suggesting that OXY didn’t have this effect during our experiments, probably due to the doses. Thus, although it has been documented OXY-mediated adverse effects, our data revealed that OXY can be used as a ergogenic agent to maintain the exercise performance at HH.

### Limitations

This study has some limitations. Our physiological experiments were performed on anesthetized preparations, which could be biased; however, the influence of volatile anesthesia has been previously documented [61], and we used α-chloralose and urethane, commonly used in chemoreflex and BR studies involving whole animal preparation [62, 63]. Our study also failed to determine the cellular and molecular mechanisms that could govern the effects of exogenous OXY administration, such as the expression of muscarinic receptors in the heart, since these receptors are involved in the parasympathetic response as well as the vasoactive intestinal peptide receptor in the heart. Furthermore, since OXY can cross the blood-brain barrier [64], it is possible that cardiovagal oxytocin neurons could be activated. Further, the SP of our results oscillates between small and medium statistical power, which could be a bias in our results due to the small sample size. Then, future research needs to consider increasing the sample size so as not to fall into a type I statistical error.

## Conclusions

High-altitude hypobaric hypoxia is known to cause short-term impairments in autonomic control, including decreased vagal outflow and chemoreflex potentiation, as well as BR withdrawal. These impairments are accompanied by large impairments in exercise performance. The role of parasympathetic modulation in physical capacity has been recognized, but the impact of OXY on exercise performance in HH was unexplored. The present study demonstrates that the chronic administration of exogenous OXY significantly improved exercise performance, the vagal response, and the BR control in HH without affecting chemoreflex function.

## Data Availability

The datasets are available from the corresponding authors upon request.
